# 586 Graft Loss: 5-year Review of a Single Burn Center’s Experience with an Institutional Grading Scale

**DOI:** 10.1093/jbcr/irac012.214

**Published:** 2022-03-23

**Authors:** Lauren B Nosanov, Taryn E Travis, Shawn Tejiram, Melissa Brantley, Melissa M McLawhorn, Lauren T Moffatt, Jeffrey W Shupp, Laura S Johnson

**Affiliations:** New York Presbyterian / Weill Cornell Medical Center, New York, New York; MedStar Washington Hospital Center, Washington, District of Columbia; MedStar Washington Hospital Center, Kensington, Maryland; MedStar Washington Hospital Center, Washington, District of Columbia; MedStar Health Research Institute, Washington, District of Columbia; Burn Center at MedStar Washington Hospital Center, Washington DC, District of Columbia; MedStar Washington Hospital Center, Washington DC, District of Columbia; MedStar Washington Hospital Center / Georgetown School of Medicine, Washington, District of Columbia

## Abstract

**Introduction:**

Despite increasing attention on provisioning quality burn care, there continues to be no consensus definition of burn “graft loss”, nor a uniformly adopted scale to grade severity. The American Burn Association Burn Quality Improvement Project metric is a binary code identifying only "failure to achieve >95% autograft take." We introduced an institutional graft loss scale in 2014 for quality improvement and reported initial results and interrater reliability. With additional data now available for analysis, a secondary review was undertaken.

**Methods:**

All patients with graft loss were identified on departmental Morbidity and Mortality (M&M) reports between 7/2016-7/2021. Graft loss grades were assigned during clinical care per institutional scale (Table 1). Non-burn acute surgical wounds that underwent skin grafting were included, but chronic non-healing wounds were excluded. Data abstracted included demographics, medical history, injury details, surgical procedures, graft loss, and lengths of stay. In situations where the graft loss grade recorded on M&M documentation was discordant with the grade documented in the medical record, the grade assigned at the later point in time was used for analysis.

**Results:**

Graft loss was noted in 260 instances for 200 patients. Mean age was 51.4(±7.5y)years. The majority were male (60.7%) and African American (48.0%). Smoking (26.0%) and diabetes (37.5%) were prevalent. Overall mortality was 4% (8/200). Graft loss percentages by grade are 28% (grade I), 28% (grade II), 13% (grade III), and 22% (grade IV). An additional 9% were deemed either unknown or due to a technical error. Overall reporting of graft loss grades improved over time as surgeons became more familiar with the scale. Reported percentages of Grades I and II graft loss also increased over time, indicating better compliance with the overall goals of this quality improvement project.

**Conclusions:**

A graft loss grading scale can be applied to track split thickness autografting outcomes among a diverse group of surgeons. Regular reporting of low grade graft loss needs to be done to understand the complete distribution of graft loss after surgical intervention, including documentation of allograft and other skin substitute loss. Improved reporting in the medical record can optimize data collection for quality assessment purposes.

